# Modeling and Design Optimization of Multifunctional Membrane Reactors for Direct Methane Aromatization

**DOI:** 10.3390/membranes7030048

**Published:** 2017-08-29

**Authors:** Nicholas J. Fouty, Juan C. Carrasco, Fernando V. Lima

**Affiliations:** Department of Chemical and Biomedical Engineering, West Virginia University, Morgantown, WV 26506, USA; nicholas.j.fouty@gmail.com (N.J.F.); jcarasco@mix.wvu.edu (J.C.C.)

**Keywords:** optimization, multifunctional membrane reactors, direct methane aromatization, natural gas utilization, modeling, design

## Abstract

Due to the recent increase of natural gas production in the U.S., utilizing natural gas for higher-value chemicals has become imperative. Direct methane aromatization (DMA) is a promising process used to convert methane to benzene, but it is limited by low conversion of methane and rapid catalyst deactivation by coking. Past work has shown that membrane separation of the hydrogen produced in the DMA reactions can dramatically increase the methane conversion by shifting the equilibrium toward the products, but it also increases coke production. Oxygen introduction into the system has been shown to inhibit this coke production while not inhibiting the benzene production. This paper introduces a novel mathematical model and design to employ both methods in a multifunctional membrane reactor to push the DMA process into further viability. Multifunctional membrane reactors, in this case, are reactors where two different separations occur using two differently selective membranes, on which no systems studies have been found. The proposed multifunctional membrane design incorporates a hydrogen-selective membrane on the outer wall of the reaction zone, and an inner tube filled with airflow surrounded by an oxygen-selective membrane in the middle of the reactor. The design is shown to increase conversion via hydrogen removal by around 100%, and decrease coke production via oxygen addition by 10% when compared to a tubular reactor without any membranes. Optimization studies are performed to determine the best reactor design based on methane conversion, along with coke and benzene production. The obtained optimal design considers a small reactor (length = 25 cm, diameter of reaction tube = 0.7 cm) to subvert coke production and consumption of the product benzene as well as a high permeance (0.01 mol/s·m^2^·atm^1/4^) through the hydrogen-permeable membrane. This modeling and design approach sets the stage for guiding further development of multifunctional membrane reactor models and designs for natural gas utilization and other chemical reaction systems.

## 1. Introduction

Large reserves of natural gas have been discovered in recent years, bringing attention to their use for hydrocarbon synthesis. According to the U.S. Energy Information Administration (EIA) [1], 26.4 trillion cubic feet of gas was produced in 2016. Estimates expect that number only to grow in the coming years as new technologies for retrieving natural gas continue to thrive and grow themselves. By 2019, it is expected that the U.S. will produce 28 trillion cubic feet per year, with 40 percent of that coming from the Marcellus shale, which includes West Virginia and much of Appalachia [2].

Utilizing the potential of natural gas comes with the intriguing issue of converting its primary hydrocarbon, methane, to higher-value chemicals. There are different methods for performing this conversion, both direct and indirect. Indirect methods usually involve the production of synthesis gas via steam reforming or other reforming methods followed by a Fischer-Tropsch process or similar process that converts the syngas to higher-order hydrocarbons. There are several direct methods, as well [3,4], such as oxidative coupling of methane (OCM) and aromatization. These methods have been of interest in recent years due to the elimination of the syngas step, as it can be a costly process. These processes are slow to occur because conversion of methane to higher hydrocarbons is thermodynamically unfavorable and often requires temperatures in excess of 1500 K to achieve a viable conversion level. Now, with advances made in catalysis and the large quantity of natural gas resources available, the direct methods have potential to become feasible.

**Figure 1:**
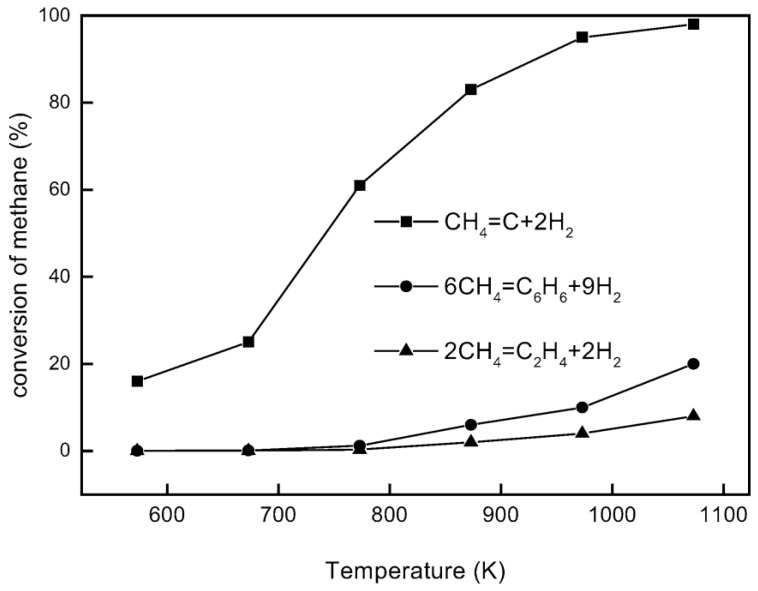
Equilibrium conversion of methane for direct conversion methods under non-oxidative conditions [6]. Copyright 2003 Elsevier.

**Figure 2:**
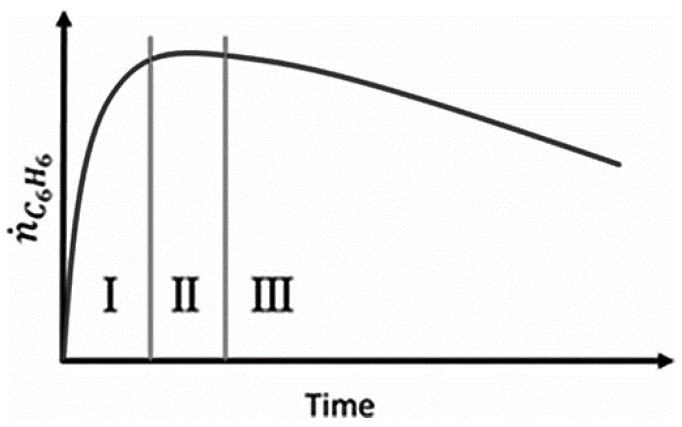
Benzene formation in typical DMA processes (I. Activation. II. Pseudo-steady state. III. Deactivation [7]). Copyright 2015 Wiley.

Aromatization of methane, or direct methane aromatization (DMA), in particular, has shown great potential, as it is thermodynamically more favorable to convert methane to aromatics directly, as opposed to olefins, as shown in Figure 1, where the equilibrium conversion to aromatics is higher than the conversion to olefins at high temperatures. When oxygen is added to a methane aromatization system, it makes the reaction of methane to benzene and hydrogen more thermodynamically favorable; however, the combustion reaction is, unfortunately, more favorable than the desired reaction, resulting in low conversions and selectivity to benzene. Wang et al. [5] first reported the non-oxidative aromatization in a fixed bed reactor using the HZSM-5 zeolite supported molybdenum catalyst (Mo/HZSM-5). For this reaction, benzene and hydrogen were produced using a pure methane feed at a high rate with minimal side products without oxygen and without combustion products. This has attracted much attention, as not only is this reaction more favorable than previous methane conversion processes, it is also environmentally conscious due to the lack of production of troublesome greenhouse gases, such as CO_2_. However, some issues with this method are the overall low conversion of methane and the rapid catalyst deactivation by coking. A general benzene production trend for the DMA process is shown over time in Figure 2 to highlight some of these issues. There have been many attempts to mitigate these issues, with solutions involving membranes, catalysis, and selective oxidation.

Considering the information presented above, the objective of this paper is to develop a detailed and comprehensive process model of the DMA reaction system while attempting to subvert the limitations presented above through membrane technology with design and process optimizations.

## 2. Literature Review

Non-oxidative DMA has emerged as a potential path for efficient natural gas utilization. However, the DMA process is limited by thermodynamic and kinetic constraints [5,7,8,9]. The overall process is endothermic and involves upgrading methane to hydrocarbons, thus leading to unrestricted carbon chain growth. All of these factors lead to low methane conversion, low benzene selectivity, and quick deactivation of the catalyst by coking. Ways to improve the process through addition of extra reactions have been reported. Addition of co-reacting alkenes and higher alkanes has been found to lower the required reaction temperature, and increase methane conversion and aromatic selectivity [10,11]. It has been reported that injection of CO, CO_2_ or water suppresses coke formation through the use of steam reforming [12,13]. Also, oxidative coupling of methane by way of small additions of oxygen to the reactor have produced greater catalyst stability with an integrated recycle system [14]. Comprehensive kinetic models based on elementary steps have been formulated for DMA [15,16]. However, none of the reported research has shown the oxidative reaction mechanisms and kinetics.

There is also an abundance of research attempting to find improved catalysts and characterize their mechanisms with DMA. Though Mo-based catalysts on zeolite supports are typically used, other transition metals have been attempted, sometimes added as promoters to molybdenum or sometimes the transition metals alone, with rhenium (Re) and tungsten (W) being the most successful alternatives [17,18]. HZSM-5 was the original and most successful of the zeolite supports, with newer cases using the MWW or MFI supports due to favorable pore size and structure [5,19,20,21]. Mo-based catalysts react to form molybdenum carbide, which is essential in the formation of aromatics from methane. Attempts to characterize the mechanisms for coking concluded that molybdenum carbide seals the channels in the zeolite support, blocking the species from reaching Brønsted acid sites (BAS) [22,23,24,25].

Membrane reactors have emerged as a potential solution to these reaction challenges. There have been many efforts involving hydrogen-selective membranes used to overcome the equilibrium barriers, leading to higher methane conversions [26,27,28]. However, it has been proven difficult to replicate predictive models in experimental work due to the lack of sufficient hydrogen permeation fluxes through the membrane and of sufficient catalyst resistances to coking, which has been accelerated due to hydrogen removal [28,29]. It has also been shown that oxygen can be used to inhibit coke formation and to decrease catalyst deactivation by using selective oxidation [30]. By way of an oxygen permeable membrane, oxygen has been shown to decrease catalyst deactivation, though as there was no hydrogen removal in the reported study, methane conversion stayed low [31]. Oxygen pulsing has also been used to combust coke deposits in the reactor in a deactivation/regeneration cycle leading to a longer lasting catalyst [32]. Furthermore, fluidized beds have been reported to show better yields than fixed beds, due to homogeneous temperatures and easier catalyst regeneration [33].

Therefore, based on the reported studies, there is a lack of systems studies that focus on mitigating both the catalyst deactivation and the equilibrium limitations. Also, there are no detailed studies attempting to model both the non-oxidative and oxidative DMA reaction mechanism, as well as the coking mechanism. This paper corresponds to a contribution aiming to fill these identified gaps.

## 3. Background

### 3.1. DMA Membrane Reactor Model

The main contributions for the DMA membrane reactor modeling have been detailed in Carrasco and Lima [34,35,36], Li et al. [26,37], and Rival et al. [28]. Those contributions have been summarized below and have been essential to the beginning of this research.

DMA is the reaction of methane (CH_4_) to hydrogen (H_2_) and benzene (C_6_H_6_). It is typically done over a molybdenum (Mo) catalyst on some zeolite support (typically HZSM-5, MWW or MFI). Equations (1)–(4) represent a two-step reaction mechanism, followed by the respective rate laws developed by Li et al. [26,37].

(1) $$\mathrm{Step}\;1:\;2{\;\mathrm{CH}}_{4}\;={\;C}_{2}H_{4}\;+\;2{\;H}_{2}$$

(2) $$\mathrm{Step}\;2:\;3{\;C}_{2}H_{4}\;={\;C}_{6}H_{6}\;+\;3{\;H}_{2}$$

(3) $$r_{1}={\;k}_{1}C_{{\mathrm{CH}}_{4}}\left(1-\frac{{\;C}_{C_{2}H_{4}}{\;C}_{H_{2}}^{2}\;}{{\;K}_{1}{\;C}_{{\mathrm{CH}}_{4}}^{2}}\right)$$

(4) $$r_{2}={\;k}_{2}C_{C_{2}H_{4}}\left(1-\frac{C_{C_{6}H_{6}}{\;C}_{H_{2}}^{3}\;}{{\;K}_{2}C_{C_{2}H_{4}}^{3}}\right)$$

in which C_i_ is the concentration in the gas phase of each i species, r_1_ and r_2_ represent the reaction rates of each reaction, k_1_ and k_2_ correspond to the respective reaction rate constants, and K_1_ and K_2_ are the equilibrium constants, determined by thermodynamic data from Yaws [38]. In the mathematical model, for a given reaction, if C_i_ = 0 for a reactant, then the corresponding reaction rate is set to zero. This conditional is implemented in MATLAB^®^ to avoid singularities in the rate denominators.

As both of the reactions are equilibrium-based, it is important to take equilibrium into account. The issue of low methane conversion, which in a fixed bed system is typically ~12% at 1000 K, is caused by high equilibrium concentrations of hydrogen, which is produced in both reactions, pushing equilibrium toward the reactants. Removal of the hydrogen from the reactor would be one alternative to overcome this limitation. A membrane reactor would fit this role well. Membrane reactors are systems that enable process intensification by combining a reactor and a separator into one unit, allowing for higher process efficiency. They typically enable higher conversions due to the selective removal of product species through the membrane. For DMA, the reaction equilibrium is shifted toward the products when hydrogen is removed via a hydrogen-selective membrane. Membrane reactors present a challenging area of research due to modeling and design challenges as well as several process target specifications that need to be achieved.

**Figure 3:**
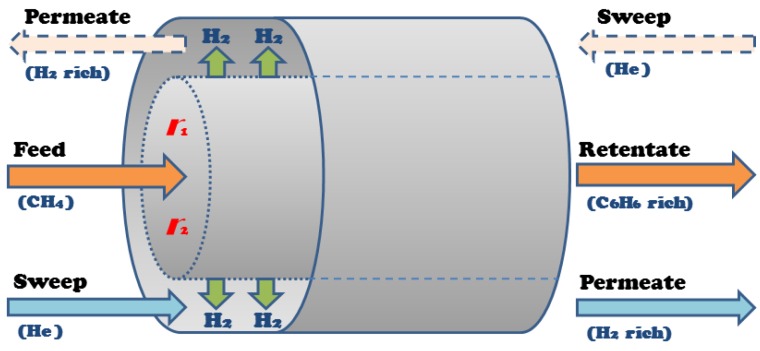
Membrane reactor co-current configuration [36].

A shell and tube membrane reactor design was considered for model development, shown in Figure 3, where methane feeds into the reactor side, the tube packed with catalysts, and the sweep gas, which in this case is helium (He), flows through the shell, or permeation side. The hydrogen produced in the tube permeates into the shell through the membrane that surrounds the wall of the tube. The tube and shell outlet streams—the retentate and permeate, respectively—are rich in benzene and hydrogen, respectively.

In Equations (5)–(12), mass balances are given for the nonlinear membrane reactor model that represents this design.

Molar balances inside tube:

(5) $$\frac{{\mathrm{dF}}_{t,{\mathrm{CH}}_{4}}}{\mathrm{dz}}\;={\;r}_{1}A_{t}\;-\;\frac{Q}{\propto_{H_{2}/{\mathrm{CH}}_{4}}\;}\left(p_{t,{\mathrm{CH}}_{4}}^{1/4}-{\;p}_{s,{\mathrm{CH}}_{4}}^{1/4}\right){\pi \;d}_{t}$$

(6) $$\frac{{\mathrm{dF}}_{t,C_{2}H_{4}}}{\mathrm{dz}}\;=\;-\frac{r_{1}}{2}A_{t}\;+{\;r}_{2}A_{t}\;-\frac{Q}{\propto_{H_{2}/C_{2}H_{4}}\;}\left(p_{t,C_{2}H_{4}}^{1/4}-{\;p}_{s,C_{2}H_{4}}^{1/4}\right){\pi \;d}_{t}$$

(7) $$\frac{{\mathrm{dF}}_{t,H_{2}}}{\mathrm{dz}}\;=\;-{\;r}_{1}A_{t}\;-{\;r}_{2}A_{t}\;-\;Q\left(p_{t,H_{2}}^{1/4}-{\;p}_{s,H_{2}}^{1/4}\right){\pi \;d}_{t}$$

(8) $$\frac{{\mathrm{dF}}_{t,C_{6}H_{6}}}{\mathrm{dz}}\;=\;-\frac{r_{2}}{3}A_{t}-\;\frac{Q}{\propto_{H_{2}/C_{6}H_{6}}\;}\left(p_{t,C_{6}H_{6}}^{1/4}-{\;p}_{s,C_{6}H_{6}}^{1/4}\right){\pi \;d}_{t}$$

Molar balances inside shell:

(9) $$\frac{{\mathrm{dF}}_{s,{\mathrm{CH}}_{4}}}{\mathrm{dz}}\;=\;\frac{Q}{\propto_{H_{2}/{\mathrm{CH}}_{4}}}\left(p_{t,{\mathrm{CH}}_{4}}^{1/4}-{\;p}_{s,{\mathrm{CH}}_{4}}^{1/4}\right){\pi \;d}_{t}$$

(10) $$\frac{{\mathrm{dF}}_{s,C_{2}H_{4}}}{\mathrm{dz}}\;=\;\frac{Q}{\propto_{H_{2}/C_{2}H_{4}}}\left(p_{t,C_{2}H_{4}}^{1/4}-{\;p}_{s,C_{2}H_{4}}^{1/4}\right){\pi \;d}_{t}$$

(11) $$\frac{{\mathrm{dF}}_{s,H_{2}}}{\mathrm{dz}}\;=\;Q\left(p_{t,H_{2}}^{1/4}-{\;p}_{s,H_{2}}^{1/4}\right){\pi \;d}_{t}$$

(12) $$\frac{{\mathrm{dF}}_{s,C_{6}H_{6}}}{\mathrm{dz}}\;=\;\frac{Q}{\propto_{H_{2}/C_{6}H_{6}}}\left(p_{t,C_{6}H_{6}}^{1/4}-{\;p}_{s,C_{6}H_{6}}^{1/4}\right){\pi \;d}_{t}$$

where F_t,i_ and F_s,i_ are the molar flow rates for each species i inside the tube and the shell, respectively. The variables z, d_t_ and A_t_ are the differential reactor length, diameter and cross-sectional area, respectively. The membrane flux expression is considered to be proportional to the membrane partial pressure gradient and has a ¼ order dependence associated with an ion-transport membrane. Q is the hydrogen permeance through the membrane, and α_H2/i_ is the selectivity between hydrogen and species i. p_t,i_ and p_s,i_ are the partial pressures of components i in the tube and shell sides, respectively. This model assumes plug flow and that the streams within each zone are well mixed. The reaction rate constant units from the original source [37] have been converted to a reactor volume basis by using the catalyst bed density in the packed bed reactor. The catalyst is packed along the length of the reaction zone with an assumed void fraction of 0.5 and catalyst effectiveness factor of 0.9. These two fractions are incorporated into the reaction rates and the cross-sectional area in the model. Also, the diameter of the catalyst particle considered between 180–425 µm is assumed small enough to neglect intraparticle transport limitations [20].

This initial model, which serves as the basis for this paper, does not consider catalyst deactivation, which will be discussed below.

### 3.2. Role of Selective Oxidation

Cao et al. [31] and Yuan et al. [30] highlighted the usefulness of selective oxidation into the non-oxidative DMA system for dealing with catalyst deactivation. Those contributions are summarized below, along with information from Tempelman et al. [23,24] describing some of the catalytic mechanisms.

The actual catalytic mechanism for DMA is still under research, but most studies agree that the mechanism for Mo-based catalysts begins with methane that goes through molybdenum carbide, where ethylene is formed, and then through the BAS on the zeolite support, where the ethylene is converted to aromatics. It has been hypothesized that coking begins when molybdenum carbide is formed within the pores of the zeolite, blocking access to the BAS for methane or keeping ethylene from exiting from the zeolite channels, forming both polyolefinic and polyaromatic cokes.

The oxidative DMA reaction is shown below in Equation (13).

(13) 6 CH4+92O2→C6H6+9 H2O

**Figure 4:**
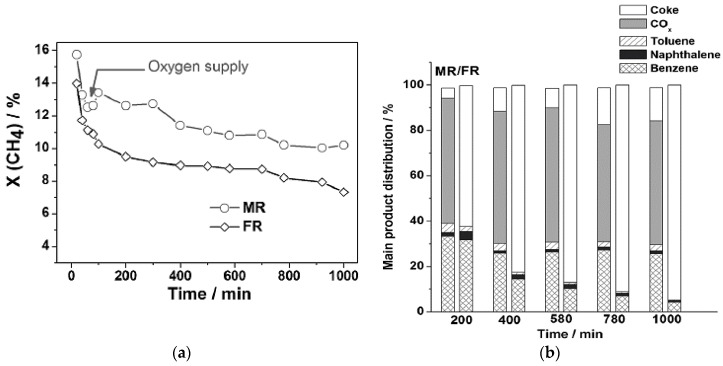
Methane conversion over time for (**a**) membrane reactor (MR) and fixed-bed reactor (FR) and (**b**) Product selectivity [31]. Copyright 2013 Wiley.

It has been shown that the presence of oxygen and steam suppresses coke formation, and stoichiometric levels of oxygen inhibit formation of molybdenum carbide [31]. By slow introduction of oxygen through an oxygen-selective membrane, the catalyst deactivated within the same time span, but to a lesser degree. Less coke was formed, but most of the gained selectivity was not toward aromatics. The results of these experiments are shown in Figure 4. It is important to notice that coke production is decreased by ~80%–90% over several hours. However, most of the carbon goes to CO*_x_* products, as opposed to benzene.

As this experiment shows, catalyst deactivation can be inhibited to a large degree by oxygen entering the system. However, this additional feed allows many more side reactions to occur, further complicating the DMA system.

## 4. Proposed Approach

The main objective in this research is to develop a model and an optimization study of a simulated DMA multifunctional membrane reactor system. The study will lead to more comprehensive models built for DMA, including the incorporation of additional reactions involving coke, naphthalene and oxygen, as well as the study of multifunctional membrane reactor designs and their performance.

### 4.1. Reaction Modeling

As shown above, the DMA system can be a complex mesh of reactions. Many studies do not include the multitude of interactions among species. The proposed reaction scheme allows for interactions between the standard non-oxidative DMA reactions with the oxidative DMA contributions and coke production. These steps are included to observe if the multifunctional membrane reactor design can help solve the issues presented above.

The non-oxidative DMA reaction scheme considered here is adapted from Li et al. [37] in which, along with Equations (1) and (2), naphthalene (C_10_H_8_) is formed from ethylene and benzene, as shown in Equation (14). Naphthalene is treated as an undesired product for the DMA system.

(14) $$C_{6}H_{6}+2C_{2}H_{4}↔C_{10}H_{8}+3H_{2}$$

Cao et al. [31] and Yuan et al. [30] both mention uncertainty in the actual schemes for the oxidative DMA reaction. They state that in the non-oxidative DMA system, ethylene formation from the molybdenum carbide may be the source of coking, so alternative mechanisms for methane to ethylene were explored. Partial oxidation is typically used to produce syngas but some steam can be formed, while an OCM mechanism can act as the alternative ethylene route in which oxygen is added to a non-oxidative DMA system. This steam can be useful for coke inhibition. The full comprehensive methane oxidation kinetic model from Stansch et al. [39] is used here to account for the oxidative DMA mechanism. This model introduces water and CO*_x_* products into the system, as well as a water-gas shift reaction step.

There are few systems studies found to determine the kinetics of the coking mechanism specifically for a DMA system, but none that give a simple, non-elementary step process model. In the developed model, it is assumed all the coking can be derived from an aromatics-to-soot pyrolysis model that includes oxygen effects, and the model from Fuentes-Cano et al. [40] is used with all relevant reactions, summarized in Equations (15)–(19). For more details on the kinetic model, refer to Fuentes-Cano et al. [40]. All new reaction species (acetylene [C_2_H_2_], pyrene [C_16_H_10_], and elemental carbon [C]) added from this reaction scheme are considered as coking products in this model.

(15) $$C_{2}H_{4}\to C_{2}H_{2}+H_{2}$$

(16) $$C_{10}H_{8}\to 0.625{\;C}_{16}H_{10}+0.875{\;H}_{2}$$

(17) $$C_{6}H_{6}\to C_{2}H_{2}+0.25{\;C}_{16}H_{10}+0.75{\;H}_{2}$$

(18) $$C_{16}H_{10}\to 16\;C+5{\;H}_{2}$$

(19) $${\mathrm{CH}}_{4}+H_{2}O↔\mathrm{CO}+3H_{2}$$

### 4.2. Membrane Modeling

**Figure 5:**
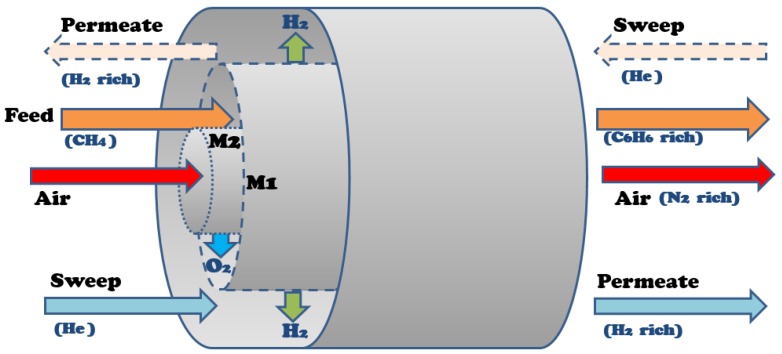
Multifunctional membrane reactor design for DMA.

The developed multifunctional membrane model employs two membranes in one system that separate oxygen from air and hydrogen from the reaction zone. Thus, there are three zones in the model based on the membrane reactor design shown in Figure 5: the reaction zone, where the feed methane is introduced and all the reactions take place; the outer shell, where helium is introduced as a sweep gas and carries separated hydrogen removed from the reaction zone via the hydrogen-permeable membrane (M1); and the inner tube, where air enters and the oxygen from the air passes through the oxygen-permeable membrane (M2) to the reaction zone.

The membrane reactor model used here for simulation and performance assessment studies is a one-dimensional, isothermal model that operates at a steady state and where all flows are co-current with respect to the feed stream. These assumptions are for a reasonable estimation of operation for a laboratory-scale membrane reactor. The basis for this model is the model presented above in Equations (5)–(12). Assuming plug flow and well-mixed flow within each zone, the membrane reactor model considers species mole balances, as summarized in Equations (20)–(22):

Mole balance, reaction zone:

(20) $$\frac{{\mathrm{dF}}_{r,i}}{\mathrm{dz}}=r_{j,i}A_{r}+J_{i,1}{\pi d}_{1}+J_{i,2}{\pi d}_{2}$$

where F_r,i_ is the flow rate of species i in the reaction zone, z is the discrete reactor length, r_j,i_ is the species reaction rate corresponding to reaction step j, A_r_ is the cross-sectional area of the reaction zone, J_i,1_ and J_i,2_ are the molar fluxes across the membrane walls of M1 and M2, respectively, and d_1_ and d_2_ are the diameters of the tubes for M1 and M2, respectively. For elemental carbon in the reactor, J_i,1_ = J_i,2_ = 0 is assumed due to its solid phase. The sign of the molar fluxes depends on the partial pressure differences, shown below in Equations (23) and (24). The sign for the species reaction rate is positive for products and negative for reactants.

Mole balances, outer shell and inner tube, respectively:

(21) $$\frac{{\mathrm{dF}}_{M1,i}}{\mathrm{dz}}={\;J}_{i,1}{\pi d}_{1}$$

(22) $$\frac{{\mathrm{dF}}_{M2,i}}{\mathrm{dz}}={\;J}_{i,2}{\pi d}_{2}$$

where F_M1,i_ and F_M2,i_ are the flow rates in the outer shell and inner tube, respectively. The resulting model can be solved using an ODE initial-value problem solver. MATLAB subroutine “ode15s” was used due to the nature of the governing rate equations that produce a stiff ODE problem. The model for M1, derived from Li et al. [41], is based on a SrCe_0.7_Zr_0.2_Eu_0.1_O_3−δ_ (SCE) ion-transport membrane. The flux through M1 is assumed to have a ¼ order dependence on partial pressure, as shown in Equation (23), in accordance with Equations (5)–(12):

(23) $$J_{i,1}=\;\frac{Q_{1}}{\alpha_{i,1}}\left(p_{r,i}^{\frac{1}{4}}-p_{M1,i}^{\frac{1}{4}}\right)$$

where Q_1_ is the permeance of hydrogen through M1, α_i,1_ is the selectivity of species i to hydrogen for M1, and p_r,i_ and p_M1,i_ are the partial pressures of each species in the reaction zone and M1, respectively.

The model for M2 is derived from Mancini and Mitsos [42] for a La_2_NiO_4__+δ_ ion-transport membrane. The flux through M2 is also assumed to have a ¼ order dependence on partial pressure, as well as a temperature dependence, as shown in Equation (24):

(24) $$J_{i,2}=\;\frac{Q_{2}}{\alpha_{i,2}}\exp\left(\frac{-B}{T}\right)\left(p_{r,i}^{\frac{1}{4}}-p_{M2,i}^{\frac{1}{4}}\right)$$

where Q_2_ is the permeance of oxygen through M2, α_i,2_ is the selectivity of species i to oxygen for M2, p_M2,i_ is the partial pressure of each species in M2, B is an effective activation energy for M2, and T is the system temperature.

### 4.3. Simulation and Optimization Setup

The reactor feed, assumed to be pure methane, and the helium sweep molar flow rates are taken from Carrasco and Lima [34]. The helium sweep and the air feeds are assumed to be pure as well. Air is fed in excess to ensure oxygen flux over the entire length of the reactor. The reactor is held at 1050 K and is assumed to be temperature controlled with the presence of a furnace. It is also assumed to have negligible pressure drop over the length of the reactor. The base case design conditions are shown in Table 1, in which Q_2_ and B are taken from Mancini and Mitsos [42]. This base case design is used to simulate and validate the use of the multifunctional membrane reactor. Coking is assumed to have no effect on the membrane transport. The oxygen flux along the length of the reactor is ~10^−2^–10^−3^ mmol/cm^2^·h leading to CH_4_/O_2_ ratios of ~100–1000:1.

The operational performance of the reactor is analyzed below with a multivariable operability-based AIS/AOS (available input set/achievable output set) mapping study, as in Carrasco and Lima [34,35,36]. Sensitivity studies are also performed to determine the largest contributing design variables in the system. In such studies, the reactor size is varied in length and diameter simultaneously, while membrane design conditions for M1 (Q_1_ and α_i,1_) are also changed to initially produce two-dimensional input analyses. The operability mapping is performed to independently analyze the effects of reactor dimensions and M1 design parameters, respectively, on the performance variables. Then all four of the above design parameters are varied to determine the best design for a DMA system via this model.

In order to determine the best design, three performance criteria are set as maximum methane conversion and benzene production rate, with minimal coking products in addition to the constraint for plug flow reactor operation. These are defined below as Equations (25)–(28), respectively:

CH_4_ conversion (X_CH4_):

(25) $$X_{\mathrm{CH}4}=\frac{{\mathrm{CH}}_{4}\;\mathrm{converted}}{{\mathrm{CH}}_{4}\;\mathrm{in}\;\mathrm{feed}}=\frac{F_{r,\mathrm{CH}4,\;\mathrm{feed}}-\left(F_{r,\mathrm{CH}4,\mathrm{end}}+F_{M1,\mathrm{CH}4,\mathrm{end}}+F_{M2,\mathrm{CH}4,\mathrm{end}}\right)}{F_{r,\mathrm{CH}4,\;\mathrm{feed}}}\times 100\%$$

C_6_H_6_ production rate (F_C6H6_):

(26) $$F_{C6H6}=F_{r,C6H6,\mathrm{end}}\;\left[\frac{\mathrm{mg}}{h}\right]$$

Coking products (C_C_):

(27) $$C_{C}=\frac{\mathrm{carbon}\;\mathrm{from}\;\mathrm{coke}\;\mathrm{products}}{\mathrm{carbon}\;\mathrm{in}\;\mathrm{feed}}=\frac{F_{C,\;\mathrm{end}}+F_{C2H2,\mathrm{end}}+F_{C16H10,\mathrm{end}}}{F_{\mathrm{CH}4,r,\;\mathrm{feed}}}\times 100\%$$

Reactor dimension specification for plug flow in reaction zone [43]:

(28) $$L/d_{h}\geq 15$$

where d_h_ is the hydraulic diameter for an annulus, defined in Equation (29) below:

(29) $$d_{h}=d_{1}-d_{2}$$

The design is optimized using a grid search method over normalized performance criteria, so that each performance criteria has equal weight in the objective function φ, as shown in Equation (30):

(30) $$\varphi =\max\left[\frac{X_{\mathrm{CH}4}}{X_{\mathrm{CH}4,\max}}+\frac{F_{C6H6}}{F_{C6H6,\max}}+\left(1-\frac{C_{C}}{C_{C,\max}}\right)\right]$$

where the max subscript denotes the maximum value observed for the corresponding performance criteria through extensive simulations. It is assumed that only the products in the reaction zone are in the scope of this study, and thus, downstream separations are not considered for simplification purposes. These separation steps may be costly especially if oxidation products permeate through the membrane. The inclusion of such separation units in a flowsheet along with the multifunctional membrane reactor will be subject of future investigation.

## 5. Results and Discussion

### 5.1. Base Case Performance Studies

The performance of the multifunctional membrane reactor is initially assessed using the design and process conditions in Table 1. First, a tubular reactor accounting for no membrane implementation is simulated in order to determine the base case results. The hydrogen-permeable membrane and the oxygen-permeable membrane are then accounted for in separate simulations to show the effects of each in terms of process improvement. Finally, both membranes are placed in tandem. All of the same governing equations are used for each case, with the permeance (Q) set to zero if a membrane is excluded from the respective case. The results of these simulations are summarized in Table 2.

In the base tubular reactor without membrane, it is shown that coke products are formed with the desired products in the operation of this system, as expected. Also, ~10 mol % of water is formed in this case. Depending on the zeolite framework used for the DMA catalyst, the zeolite’s catalytic integrity may be compromised. Integrity issues under the steam environment have not been observed in the short term for MWW-structure zeolites [44], for which future studies will be explored based on the catalyst reported in Reference [20].

Whenever M1 is added to the system, the methane conversion increases dramatically, but the coking effects also increase by a similar factor. The conversion increase is largely due to the removal of hydrogen in the system, shifting the DMA equilibrium as described above. The coking effects increase due to increases in benzene and ethylene production, both of which contribute to the pyrolysis model in Fuentes-Cano [40].

When M2 is added to the tubular reactor system, the conversion increases slightly, while the coking effects decrease by ~10% when compared to the base case. This fact is consistent with the reported role of oxygen in coking inhibition for the DMA system. This inhibition would potentially be higher if the dynamic operation of the reactor was considered. Benzene production also decreases due to the carbon usually reserved for benzene going to other sources, such as CO*_x_* products. The benzene production loss could be an issue with this design, but a cost analysis would need to be done to compare the tradeoffs with the reduction in coking effects.

When both membranes are added to the base case tubular system, the coking effects decrease by less than ~10% when compared to the case with M1 only, similar to the effect observed when M2 was added to the tubular system without membrane. The conversion again increases dramatically when compared to the base case and the scenario with M2 only. These results show that the two effects of increased conversion and coking inhibition have potential to be enhanced in a single system to different degrees depending on the performance criteria of interest.

### 5.2. Sensitivity Studies

In order to analyze each individual effect of the design variables and determine the variables with the greatest contributions to the performance criteria, sensitivity studies are performed. Table 3 shows the ranges for each variable considered in these studies. The maximum permeances in the ranges for both membranes are assumed as the maximum values reported in their respective sources [41,42], which are based on experimental studies. Potential integrity issues at such maximum values associated with low membrane thicknesses are not accounted for in this study. Both membranes can be highly selective due to their ionic nature, and in this model, it is assumed that M2 is completely selective to oxygen, as stated in Mancini and Mitsos [42]. The selectivity of the other membrane M1, α_i,1_, is considered to be a design variable.

**Figure 6:**
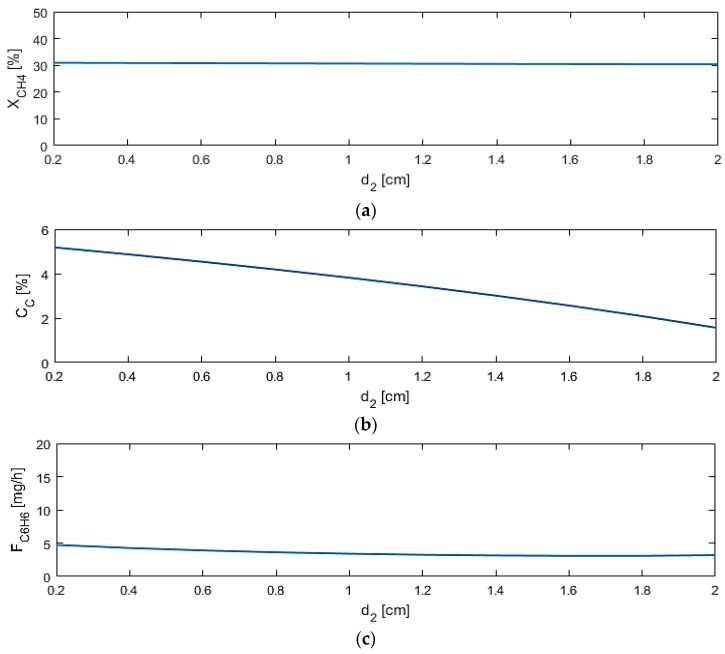
Sensitivity studies of d_2_ for model performance criteria: (**a**) X_CH4_; (**b**) C_C_; (**c**) F_C6H6_.

The reactor length (L), along with d_1_, Q_1_, and α_i,1_ significantly affect all three performance criteria, while M2 permeance (Q_2_) and diameter (d_2_) primarily affect the coke products. As only d_2_ significantly affects the coking products, shown in Figure 6, Q_2_ is assumed to be 1.3 × 10^−3^ mol/s·m^2^·atm^1/4^ as in the base case. As d_2_ increases, which causes a larger oxygen molar flux through the membrane, the coking effects decrease, which confirms that the more oxygen that is in the system, the less coke products will be produced. Due to low effects of this variable on the other performance criteria, the low, fixed value of 0.25 cm is chosen for d_2_ for the rest of the studies. This low d_2_ value is chosen to allow for d_1_ to change according to the range in Table 3.

**Figure 7:**
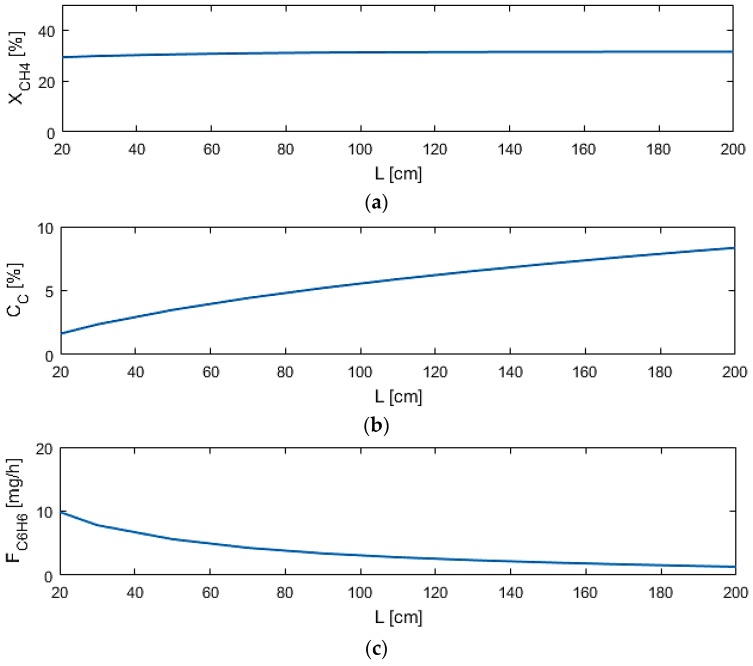
Sensitivity studies of L for model performance criteria: (**a**) X_CH4_; (**b**) C_C_; (**c**) F_C6H6_.

Reactor length (L) shows to be the most significant design variable among those selected, shown in Figure 7, as coke production ratio can reach nearly 10% at long lengths, while benzene production is hindered as well at long lengths. This is due to the DMA reactions reaching equilibrium within a short distance in the reactor. The coking effects increase and the benzene production is inhibited with length due to the increased volume for pyrolysis and benzene permeation through M1. This result indicates that the reactor design should be short in length to take both factors into account.

**Figure 8:**
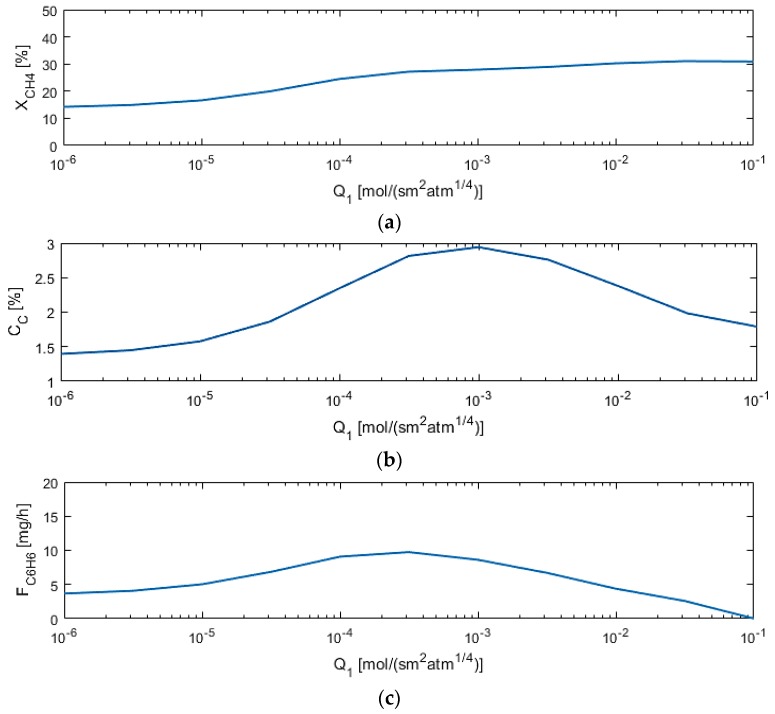
Sensitivity studies of Q_1_ for model performance criteria: (**a**) X_CH4_; (**b**) C_C_; (**c**) F_C6H6_.

M1 permeance (Q_1_) affects the methane conversion the most when compared to other parameters, as depicted in Figure 8, as expected, because the more hydrogen flows out of the reaction zone, the more the equilibrium shifts toward the products. However, too high of a permeance allows for benzene permeation out of the reaction zone.

Only three of the six sensitivity studies performed are shown in this paper due to their higher degree of importance. Due to their significant effects on the three performance criteria, reactor length as well as diameter, permeance, and selectivity for M1 are chosen for future studies to be able to determine the best multifunctional membrane reactor design.

### 5.3. Optimization and Operability Mapping

Operability mapping studies follow a region of input variables in the AIS in order to determine the best reactor design based on the performance criteria defined for the output variables in the AOS. The best point can then be selected from the output region using the optimization method described above. The ranges of variables for the AIS are decreased here from the base case in order to more finely observe such variable effects.

**Figure 9:**
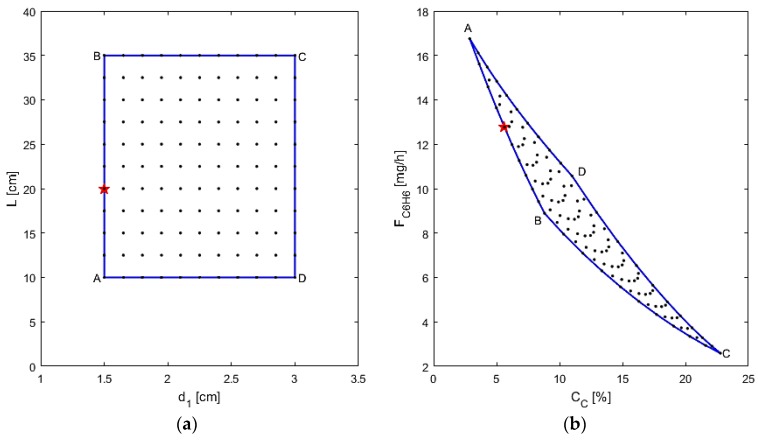
(**a**) AIS—reactor dimensions; (**b**) AOS—C_C_ and F_C6H6_.

First, the performance is assessed when varying the reactor dimensions (reactor length and M1 diameter), using Q_1_ = 0.01 mol/s·m^2^·atm^1/4^ and α_i,1_ = 1000, chosen as typical estimates of membrane parameters, as shown in Figure 9. In particular, from points “A” to “B,” as the length increases, the coking effects also increase and the benzene production decreases due to pyrolysis and benzene permeation as reported above. From points “B” to “C” the diameter is increased, so more cross-sectional area for the DMA reaction, along with all the other reactions, is present to take place in. Thus, the coking effects dramatically increase with the larger area and benzene is consumed in the process. The best design based on the optimization for these cases is determined to be a small reactor (d_1_ = 1.65 cm, L = 5 cm) with an L/D ≈ 3. This L/D is far too small for the plug flow assumption of L/D > 15 [43], and, therefore, not a realistic solution. Whenever this constraint is applied, a new optimal design is obtained, denoted by the star point in Figure 9 (d_1_ = 1.5 cm, L = 20 cm). These results are interesting because they demonstrate the flexibility of the formulated optimization problem and the variability of the optimal result depending on the incorporated process constraints. Thus, this means that the best design based on the objective function in Equation (30) calls for a short reactor with a total volume just large enough to allow for the DMA reactions to reach equilibrium.

**Figure 10:**
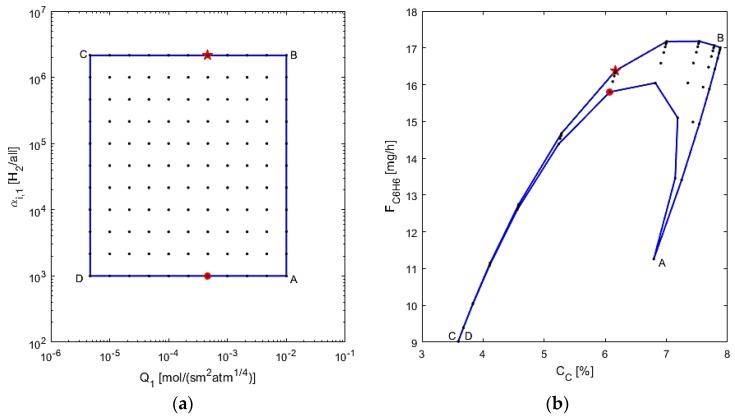
(**a**) AIS—M1 permeance and selectivity to hydrogen; (**b**) AOS—C_C_ and F_C6H6_.

The membrane design is assessed next, by varying Q_1_ and α_i,1_ and using L = 20 cm and d_1_ = 1.5 cm, chosen from the previous study’s optimal point, as depicted in Figure 10. In this figure, from points “A” to “B”, the membrane selectivity is increased, which leads to a sharp increase in benzene production and a small increase in coking effects. This is due to less benzene leaving the reaction zone, possibly allowing for more benzene and other reaction products to be converted to coke. In segment “B” to “C”, the coking effects decrease possibly because more hydrogen is left in the reaction zone due to low permeance. The benzene production decreases because there is less conversion when permeance is low, again due to more hydrogen in the reaction zone. In the “C” to “D” segment in the AIS, selectivity is decreased, but interestingly, the changes in the AOS are negligible, highlighting that at low permeance, selectivity has little effect on the performance criteria. From point “D” back to “A” it is observed that the benzene production first increases sharply, then it decreases while the permeance increases. This makes sense, since at a low selectivity, increasing permeance allows for more hydrogen to pass through the membrane, thus leading to higher conversion; however, the permeance eventually gets too large for benzene and its reactants to flow through the membrane, thus losing production overall. Hence, there is a sensitive balance at work with the permeance at low selectivity. The optimal design can thus be determined to have a membrane to allow for a mid-range to high permeance with a high selectivity, denoted by the star point (Q_1_ = 5 × 10^−4^ mol/s·m^2^·atm^1/4^, α_i,1_ = 2 × 10^6^). As it may be difficult to achieve a very high selectivity in laboratory conditions, it is suggested that the best design has a lower selectivity as an alternative for a mid-range permeance, as the optimal results in this case would change by <10%, seen at the red circle point (Q_1_ =5 × 10^−4^ mol/s·m^2^·atm^1/4^, α_i,1_ = 10^3^). The performed operability mapping thus also allows for determining alternative optimal designs for the membrane reactor system.

**Figure 11:**
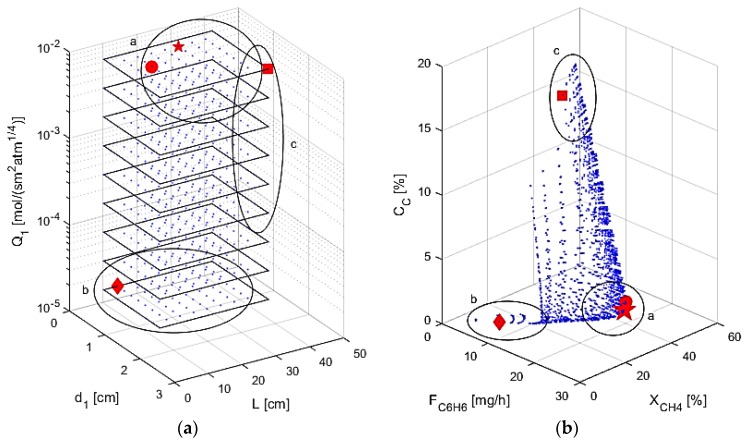
(**a**) AIS—reactor dimensions and M1 permeance; (**b**) AOS—performance criteria.

In the final study completed, all four of the above design parameters are varied, as shown in Figure 11. Selectivity, α_i,1_, is not considered in the figure for graphical purposes and due to the fact that the optimal designs always had the maximum selectivity. Table 4 shows the optimal designs obtained within the range simulated, as well as the optimal performance criteria for different cases considering higher weights for specific performance criteria. Case 1 uses the objective function φ shown in Equation (30), while Cases 2–4 use 100 times weight toward benzene production, methane conversion, and coke production, respectively. The considered ranges are similar to the ones used in the mapping studies with two inputs. The maximum/minimum range values assumed for some of the inputs are also presented in Table 4 to account for membrane properties that would be more feasible in a laboratory setting. As noted before, high permeance and high selectivity allows for the most hydrogen to leave the system, allowing for increased conversion of methane, while also allowing for the desired benzene to stay in the reaction zone. A small reactor is shown to give the lowest coke formation, but the reactor must be large enough to convert the feed and still be considered a plug flow reactor. Given these considerations, zone “a” in Figure 11 shows the optimal region: high permeance and selectivity with small reactor dimensions. This zone contains the best designs according to the objective function for Cases 1 and 2, denoted by the large star and the circle, respectively. Cases 1 and 2 have very similar results, showing that high benzene production coincides with low coke production and high conversion. The zone “b” shows the region with small reactor dimensions with low permeance, not allowing for the equilibrium of the DMA reactions to be shifted toward the products through hydrogen removal. The optimal point for Case 4 is contained in zone “b”, denoted by the diamond point, and since permeance is low, the reactor acts as a tubular reactor with no M1, only M2, such as in the base case simulations above. The criteria are at very low values due to low reaction volume, so minimal coking also coincides with little feed reacting. Region “c” is where larger reactor dimensions are used with high permeance, highlighted by the large amount of coke production that occurs. The best design for Case 3 is in this zone, denoted by the square point, showing that maximal conversion can also lead to high coke production and low benzene production. This is due to the large reactor dimensions allowing for pyrolysis and benzene permeation as explained above. The optimization studies performed thus demonstrate that this multifunctional membrane reactor for a DMA system should have a small volume to inhibit the coking effects and a high permeance through M1 for conversion enhancement.

## 6. Conclusions

A detailed and comprehensive multifunctional membrane reactor model was developed for direct methane aromatization (DMA) that accounts for both oxidative and non-oxidative mechanisms and for coke production. A reaction model was formulated using non-oxidative DMA, oxidative coupling of methane, and aromatics-to-soot pyrolysis reaction mechanisms. This reaction model was then further developed by adding a multifunctional membrane model that allowed for oxygen to permeate from an air feed through an oxygen-permeable membrane to the reactor and for hydrogen to permeate through a separate hydrogen-permeable membrane out of the reactor along its length. The hydrogen-permeable membrane is added to drive the equilibrium toward the products for the DMA reaction system. The oxygen-permeable membrane allows for a slow flow of oxygen to enter the DMA system, as previous studies have indicated that the oxygen inhibits coke formation in the reactor. Performance criteria were defined to maximize methane conversion and benzene production, as well as minimize coke production for this DMA system. These performance criteria were used to determine the optimal reactor design given the process conditions by using an objective function. Results showed that the optimal reactor design calls for a reactor short in length (25 cm), and relatively narrow in diameter (0.7 cm), to minimize the coking effects on this system. The optimal hydrogen-permeable membrane design was determined to require a high permeance (0.01 mol/s·m^2^·atm^1/4^) and a high selectivity (greater than 10^5^) to hydrogen. If a high selectivity cannot be achieved, a high permeance is best to allow for hydrogen to be removed and shift the equilibrium toward the products. The optimization and operability mapping performed allowed the determining of feasible ranges of expectations for outputs when further developing this system.

Using this design and model developed, it would be possible to explore how process conditions of feed and sweep molar flow rates, feed compositions accounting for co-fed systems, temperature, pressure, and the oxygen-permeable membrane design could affect the DMA reactor system. The inclusion of such conditions in the optimization problem formulation is currently under investigation. This will allow for future insights into the process development of more comprehensive and larger-scale DMA systems. This process systems approach has the potential to guide further development of multifunctional membrane reactor models and designs for natural gas utilization systems and other chemical reaction systems.

## Figures and Tables

**Table 1:** Base case simulation design and process conditions.

Parameter (Unit)	Value	Parameter (Unit)	Value
Temperature (K)	1050	d_2_ (cm)	0.5
Pressure (atm)	1	Q_1_ (mol/s·m^2^·atm^1/4^)	0.01
F_CH4,feed_ (mmol/h)	4.98	Q_2_ (mol/s·m^2^·atm^1/4^)	1.3 × 10^−3^
F_air,feed_ (mmol/h)	23.8	α_i,1_ (H_2_/all)	10^6^
F_He,sweep_ (mmol/h)	6.24	α_i,2_ (O_2_/all)	10^6^
L (cm)	25	B (K)	10,240
d_1_ (cm)	1.25	–	–

**Table 2:** Base case performance criteria results. Base uses no membrane, M1 uses H_2_-permeable membrane, M2 uses O_2_-permeable membrane, and multifunctional uses both membranes.

Output (Unit)	Base	M1	M2	Multifunctional
X_CH4_ (%)	19.52	38.36	19.57	38.15
F_C6H6_ (mg/h)	10.49	19.85	8.65	18.09
C_C_ (%)	2.28	4.95	2.06	4.64

**Table 3:** Sensitivity study input variable ranges.

Input (Unit)	Range
L (cm)	20–200
d_1_ (cm)	0.5–3
d_2_ (cm)	0.2–2
Q_1_ (mol/s·m^2^·atm^1/4^)	10^−6^–0.1
Q_2_ (mol/s·m^2^·atm^1/4^)	10^−7^–10^−2^
α_i,1_ (H_2_/all)	10^2^–10^7^

**Table 4:** Optimal reactor designs and outputs with modified objective functions: Case 1 has equally weighted criteria, as seen in φ; Case 2 has 100 times weight on F_C6H6_; Case 3 has 100 times weight on X_CH4_; Case 4 has 100 times weight on C_C_.

Input/Output (Unit)	Case 1	Case 2	Case 3	Case 4
L (cm)	25	13	37	9
d_1_ (cm)	0.7	1.1	2.1	0.5
Q_1_ (mol/s·m^2^·atm^1/4^)	0.01 *	0.01	0.01	2.15 × 10^−5 #^
α_i,1_ (H_2_/all)	4.64 × 10^5^ *	4.64 × 10^5^	1000 ^#^	4.64 × 10^5^
F_C6H6_ (mg/h)	20.66	20.88	5.22	5.97
X_CH4_ (%)	37.82	38.18	42.17	13.06
C_C_ (%)	1.30	1.99	15.32	0.064

*/^#^ Maximum/minimum value in simulated range considered.
